# Anti-Stokes fluorescence excitation reveals conformational mobility of the C-phycocyanin chromophores

**DOI:** 10.1063/4.0000164

**Published:** 2022-09-02

**Authors:** Georgy V. Tsoraev, Elena A. Protasova, Elizaveta A. Klimanova, Yury L. Ryzhykau, Alexander I. Kuklin, Yury S. Semenov, Baosheng Ge, Wenjun Li, Song Qin, Thomas Friedrich, Nikolai N. Sluchanko, Eugene G. Maksimov

**Affiliations:** 1 Faculty of Biology, Lomonosov Moscow State University, Moscow 119991, Russia; 2 Research Center for Molecular Mechanisms of Aging and Age-Related Diseases, Moscow Institute of Physics and Technology, Dolgoprudny 141700, Russia; 3 Frank Laboratory of Neutron Physics, Joint Institute for Nuclear Research, Dubna 141980, Russia; 4 China University of Petroleum (Huadong), College of Chemical Engineering, Qingdao 266580, People's Republic of China; 5 Yantai Institute of Coastal Zone Research, Chinese Academy of Sciences, Yantai 264003, People's Republic of China; 6 Technical University of Berlin, Institute of Chemistry PC 14, D-10623 Berlin, Germany; 7 A.N. Bach Institute of Biochemistry, Federal Research Center of Biotechnology of the Russian Academy of Sciences, Moscow 119071, Russia

## Abstract

The structural organization of natural pigment-protein complexes provides a specific environment for the chromophore groups. Yet, proteins are inherently dynamic and conformationally mobile. In this work, we demonstrate the heterogeneity of chromophores of C-phycocyanin (C-PC) from *Arthrospira platensis*. Part of the population of trimeric C-PC is subject to spontaneous disturbances of protein–protein interactions resulting in increased conformational mobility of the chromophores. Upon fluorescence excitation in the visible range, the spectral signatures of these poorly populated states are masked by bulk chromophore states, but the former could be clearly discriminated when the fluorescence is excited by near-infrared quanta. Such selective excitation of conformationally mobile C-PC chromophores is due to the structure of their S_1_ level, which is characterized by a significantly broadened spectral line. We demonstrate that the anti-Stokes C-PC fluorescence is the result of single-photon absorption. By combining spectral and structural methods, we characterize four distinct states of C-PC chromophores emitting at 620, 650, 665, and 720 nm and assigned the fast component in the anti-Stokes fluorescence decay kinetics in the range of 690–750 nm to the chromophores with increased conformational mobility. Our data suggest that the spectral and temporal characteristics of the anti-Stokes fluorescence can be used to study protein dynamics and develop methods to visualize local environment parameters such as temperature.

## I. INTRODUCTION

Phycobiliproteins represent one of the most widespread classes of water-soluble proteins. Various phycobiliproteins are part of the cyanobacterial light-harvesting antenna comprising up to 50% of the dry weight of the cell.^1^ The high quantum yield of fluorescence of these proteins not only ensures their efficient operation as part of photosynthetic apparatus^2–6^ but also explains their relevance in biomedical applications in which their bright fluorescence signal is used for indication and labeling in flow-cytometry and immuno(histo)chemistry.^7,8^

**FIG. 1.**
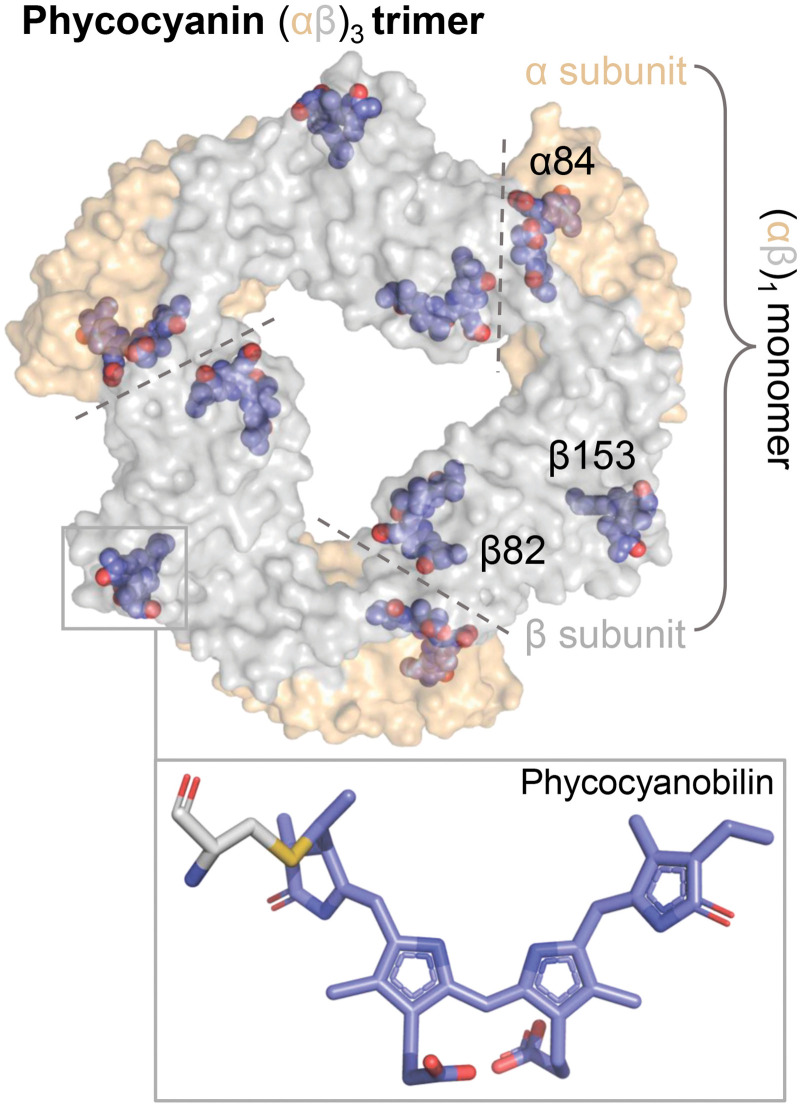
Structure of a phycocyanin (αβ)_3_ trimer (PBD ID 1GH0) and the phycocyanobilin (PCB) chromophore. Surfaces of the α-subunit (whey) and the β-subunit (gray) were rendered semitransparent to reveal all nine phycocyanobilin chromophores (shown in violet-blue), which are mostly covered by protein matrix. The phycocyanobilin chromophores are covalently bound to the protein via highly conserved cysteines at 84 position in the α-subunit and 82 and 153 in the β-subunit.

All phycobiliproteins share similarities of their structural organization. Heterodimers composed of α and β subunits form so-called monomers (or αβ_1_) of phycobiliproteins.^9,10^ Under physiological conditions, αβ monomers of different phycobiliproteins spontaneously form ring-like trimers (allophycocyanin) and hexamers (phycocyanin, PC, Fig. 1).^2,11–13^ The pigment phycocyanobilin has a tetrapyrrole nature and is the same in phycocyanin and allophycocyanin, but the spectral characteristics of these proteins differ significantly. Each αβ_1_ protomer of PC covalently binds three identical phycobilins (PCB) via cysteine residues; however, the unique protein structure provides a specific environment for the chromophore, which results in spectral tuning.^14^ The main reasons for the change in the optical properties of the protein-bound chromophore are: first, the change in the effective conjugation length of the π-electron system of linear tetrapyrrole; second, the energetic coupling of neighboring pigments; and third, the local effects of the charge distribution in the surrounding protein matrix. It should be noted that the protein does not cover all chromophores completely; some remain exposed to the solvent.^15–17^ All these effects contribute to the specific broadening of the absorption spectrum of the pigment-protein complexes, which is necessary for the effective absorption of photons and subsequent excitation energy transfer from blue to red pigments inside the phycobilisome antenna, and further to the chlorophylls of the photosynthetic reaction centers.^3,11,18–23^ It should be noted that linear tetrapyrroles are widespread not only among antenna proteins but also among photosensitive proteins because of their ability to photoisomerize, and currently being actively investigated for the development of molecular tools.^24–26^

Phycobiliproteins and phycocyanin (PC) in particular are well studied.^27^ For these proteins, the spatial structure is known and is characterized by a considerable stability and a number of similarities between different representatives of this class of proteins. However, the available crystal structures reflect only some of the possibilities for organizing the electronic levels by the conformationally mobile chromophores. Under physiological conditions, phycobiliprotein chromophores (as well as other fluorescent proteins in general) are characterized by their isomerization state reflecting different configurations of the double bonds. This property leads to heterogeneity in the spectral characteristics of the ensemble of pigment-protein complexes. This heterogeneity introduces ambiguity in the interpretation of some experimental results, such as measurements of fluorescence quantum yield, lifetime changes, FRET efficiency, etc. Evidence of the conformational mobility was also found by single-molecule spectroscopy.^28,29^ Conformational heterogeneity may reflect different functional states of the protein and the interaction of chromophores with their microenvironment. However, there is still poor understanding of the mechanisms behind the heterogeneity, and the demands of biomedical applications lead to an incessant search for variants of phycobiliproteins (and fluorescent proteins in general) with increased stability, which is, in particular, achieved via cross-linking of protein subunits.^30^

In this work, we tried to improve our understanding of the nature of the spectral characteristics of C-PC and the heterogeneity thereof using picosecond-resolved spectrofluorimetry. For this purpose, we decided to use long-wave excitation with low-energy quanta, as we expected to see the spectral signatures of the chromophores which interact most actively with the environment.

## II. MATERIALS AND METHODS

### A. Protein expression and purification

The C-PC sample was purified from *Arthrospira platensis* (*Spirulina*) as described previously.^31^ Briefly, algae cells were harvested by centrifugation and resuspended in 2 mM phosphate buffer (pH 7.0, containing 4 mM sodium azide) and then frozen and thawed three times. After centrifugation at 20* *000 g for 5 min at 4 °C, the supernatant was taken. (NH_4_)_2_SO_4_ was added to a final concentration of 1.25 M and centrifuged again to obtain a crude extract of C-PC, which was then loaded onto a pre-equilibrated Macro-Prep Methyl hydrophobic chromatographic column. The column was then washed with phosphate buffer containing 1.25, 0.9, 0.5, and 0.2 M (NH_4_)_2_SO_4_. The eluent with purity (A620/A280) greater than 4.0 was collected and concentrated with a 30 kDa ultrafiltration device. The buffer of the collected sample was changed into 0.6 M phosphate buffer (pH 8.0) and further purified using sucrose density gradient centrifugation with a linear gradient of 0.2–0.5 M in an ultracentrifuge Beckman Coulter l-100× (USA) for 16 h at 43 000 rpm at 4 °C. This preparation was then lyophilized for long-term storage. Finally, the C-PC sample (12 ml) was loaded on a Superdex 200 26/600 column (GE Healthcare) equilibrated with a 20 mM Tris-HCl pH 7.6 buffer containing 150 mM NaCl and operated at a 2 ml/min flow rate. C-PC fractions were identified by SDS-PAGE^32^ and diode-array detection in the 240–900 nm spectral range. The fractions of the main peak were pooled and analyzed by SDS-PAGE, which indicated the presence of two bands with apparent *MW* of 16 and 18 kDa, tentatively corresponding to α and β C-PC chains.

### B. Size-exclusion spectrochromatography (SEC)

Section with diode-array detection was used to analyze size and spectral properties of С-PC simultaneously. To study the effect of urea, a С-PC sample (100 *μ*l) was first dialyzed overnight against 50 ml of buffer containing 8 M urea and 30 mM β-mercaptoethanol and then clarified by centrifugation for 10 min at 4 °C and 15 600 g. Native С-PC or С-PC dialyzed against 8 M urea (50 *μ*l) was loaded on a Superdex 200 Increase 5/150 column (GE Healthcare, Chicago, Illinois, USA) pre-equilibrated with 75 mM phosphate buffer pH 7.9 and operated using a Varian ProStar 335 system (Varian Inc., Melbourne, Australia) at a 0.45 ml/min flow rate. During the runs, absorbance in the 240–900 nm range was recorded with 1-nm steps (4 nm slit width) and 1.3 Hz frequency. Diode-array data were converted into .csv files using a custom-built Python script and processed into contour plots using Origin 9.0 (Originlab, Northampton, MA, USA). Apparent molecular masses of С-PC samples were determined from the maxima of their chromatographic peaks by calibration of the column using α-lactalbumin monomer (15 kDa), BSA monomer (66 kDa), BSA dimer (132 kDa), and BSA trimer (198 kDa).

### C. Steady-state absorption spectroscopy

Steady-state absorption spectra were recorded as described earlier.^33^ The temperature of the sample was stabilized by a Peltier-controlled cuvette holder Qpod 2e (Quantum Northwest, USA) equipped with a magnetic stirrer.

### D. Time-resolved fluorescence and anisotropy measurements

Spectrally resolved fluorescence decays were recorded by a time-correlated single-photon counting (TCSPC) system SPC-150 equipped with a HPM-100–07C detector (Becker & Hickl, Germany). A ML-44 monochromator (Solar LS, Belarus) was installed in front of the detector to tune the detection wavelength from 600 to 750 nm in 5 nm steps. To record time-integrated fluorescence spectra in the FIFO mode of the SPCM64 software (Becker & Hickl, Germany), a monochromator tuning step of 1 nm was used. Stokes fluorescence^34^ was excited at 570 nm (repetition rate 80 MHz and pulse width 150 fs) using the second harmonics of a femtosecond optical parametric oscillator TOPOL-1050-C (Avesta Project LTD, Russia) pumped by a femtosecond Yb-laser (TEMA-150, Avesta Project LTD). Anti-Stokes fluorescence was excited at 770  and 780 nm (repetition rate 80 MHz, pulse width 150 fs, and optical power up to 685 mW) using the signal output wavelength of the femtosecond optical parametric oscillator. Optical power was attenuated by a set of neutral density filters (Thorlabs, USA). The laser beam was defocused on the sample to a diameter of about 0.5 cm to avoid two-photon excitation and other non-linear optical effects. The choice of such wavelengths is due to the requirement to record the fluorescence spectrum in the range from 600 to 750 nm without distortion due to laser beam scattering.

The fluorescence emission was collected perpendicular to the excitation beam. To avoid laser scattering detection, edgepass filters (Thorlabs, USA) were utilized: a long-pass 600 nm filter for Stokes fluorescence and a short-pass 750 nm filter for anti-Stokes fluorescence. The temperature of the sample during the recording of spectrally resolved fluorescence decays and fluorescence spectra was stabilized at 25 °C by a cuvette holder Qpod 2e with a magnetic stirrer (Quantum Northwest, USA). The temperature dependence measurement of anti-Stokes fluorescence spectra was carried out using the Qpod 2e system from 5 up to 60 °C with steps of 5 °C.

To measure the kinetics of fluorescence anisotropy, we used a filter-based system with a set of two ultra-broadband wire-grid polarizers WP25M-UB (Thorlabs) mounted into motorized rotation mounts K10CR1/M (Thorlabs, USA). Fluorescence decay kinetics I(t) were measured at different positions of the emission polarizer—in parallel ( ∥) and perpendicular ( ⊥) orientation to the excitation polarizer, and the anisotropy kinetics r(t) was calculated as

$$r\left(t\right)=\frac{{I(t)}_{∥}-{I(t)}_{⊥}}{{I(t)}_{∥}+2\cdot {I(t)}_{⊥}}.$$

Analysis of fluorescence and anisotropy decay curves was performed by SPCImage (Becker & Hickl, Germany). Analysis of the fluorescence attenuation kinetics revealed multiple components, but in some cases, for better presentation, we provide average fluorescence lifetimes calculated as the sum of the fractional amplitude-weighted lifetimes. The global analysis of spectrally resolved fluorescence decays was preformed using the Glotaran Software 1.5.1^35^ (Biophysics Department of the Vrije Universiteit Amsterdam). The post-processing and visualization of calculated data were performed using an Origin Pro 2018 (OriginLab Corporation, USA).

### E. Small angle x-ray scattering

SAXS experiments were performed at a Rigaku MicroMax-007HF instrument at the Moscow Institute of Physics and Technology (Dolgoprudny, Russia).^36,37^ The Rigaku SAXS instrument was equipped with a pinhole camera attached to a rotating anode x-ray high-flux beam generator (MicroMax-007HF) operating at 40 kV and 30 mA (1200 W). The samples were placed at a distance of 2.0 m from a multiwire gas-filled detector Rigaku ASM DTR Triton 200 (the active area diameter is 20 сm and the pixel size is ∼260 *μ*m). The covered *q*-range was 0.007–0.215 Å^−1^ (*q* = 4π sin θ/λ, where *λ* is the wavelength and 2θ is the scattering angle). Procedures of primary data treatment (azimuthal integration of the 2D images, *q*-calibration,^38^ and normalization of the data to transmission and path length) were performed using the Saxsgui software (Rigaku Innovative Technologies, Inc. and JJ x-ray System Aps).

SAXS data were processed using the ATSAS software suite.^39^ Primary manipulations with 1D scattering profiles (averaging, subtraction, and merging) were performed using PRIMUSqt. Pair distance distribution functions *P*(*r*) and corresponding regularized intensity profiles *I*(*q*) were calculated using the GNOM program. The GASBOR program was used for *ab initio* reconstruction of the protein structure as a chain-like ensemble of dummy residues.^40^

For the C-PC protein in 8 M urea solution, there was a significant decrease in intensity at the zero angle *I*(0) compared to standard conditions (Table S1), indicating a decrease in the oligomeric state (that is a common phenomenon for the proteins at non-native conditions). The degree of oligomer dissociation^41,42^ and/or conformational flexibility^43^ could be characterized by SAXS/SANS. Molecular weight (*MW*) of C-PC at 150 mM NaCl was calculated as *V*_c_^2^/123.1 *R*_g_ according to Ref. 44. The obtained value of 124 kDa was in good agreement with the expected *MW* of a trimer (107 kDa). *MW* estimation methods based on the calculation of Porod volume may give incorrect results in the case of partially unfolded proteins, since they have an atypical area/volume ratio. So, in the case of C-PC at 8 M urea, we estimated the *MW* in relative units according to the expression from the work,^45^ modified to take into account the difference in contrasts,

$$\frac{{MW}_{2}}{{MW}_{1}}=\frac{I_{2}\left(0\right)/\left(c_{2}\Delta \rho_{2}^{2}\right)}{I_{1}\left(0\right)/\left(c_{1}\Delta \rho_{1}^{2}\right)},$$

where *c_i_* is a protein concentration, Δ*ρ_i_* is a scattering length density (SLD) contrast, and *i* = 1 or 2 for C-PC at 150 mM NaCl or 8 M urea, respectively. The obtained *MW* ratio is equal to *MW*_2_/*MW*_1_ = 0.645 ± 0.008.

For other details of SAXS experiment and data treatment, see Table S1, which is prepared in accordance with Refs. 46 and 47. The SAXS data were deposited to SASBDB (http://sasbdb.org)^48^ with accession codes SASDPA4 and SASDPB4 for the C-PC at 150 mM NaCl and 8 M urea, correspondingly.

## III. RESULTS AND DISCUSSION

**FIG. 2.**
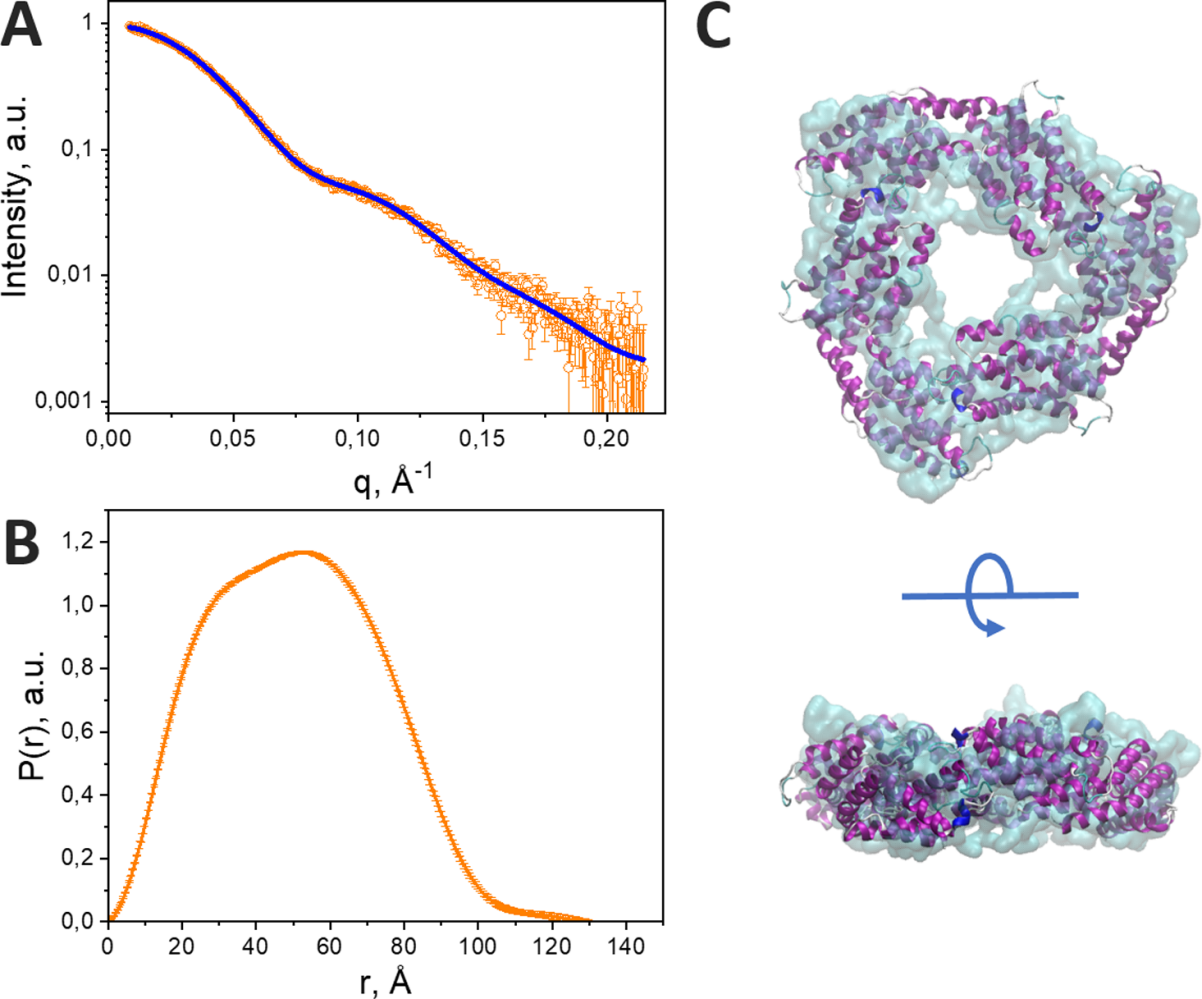
Solution structure of C-PC as revealed by SAXS. (a) *I*(q) profile (orange circles) and GASBOR approximation (blue line). (b) Pair distance distribution function. (c) Comparison of chain-like *ab initio* low-resolution structure and high-resolution structure of the C-PC trimer [PDB ID: 1gh0].

**FIG. 3.**
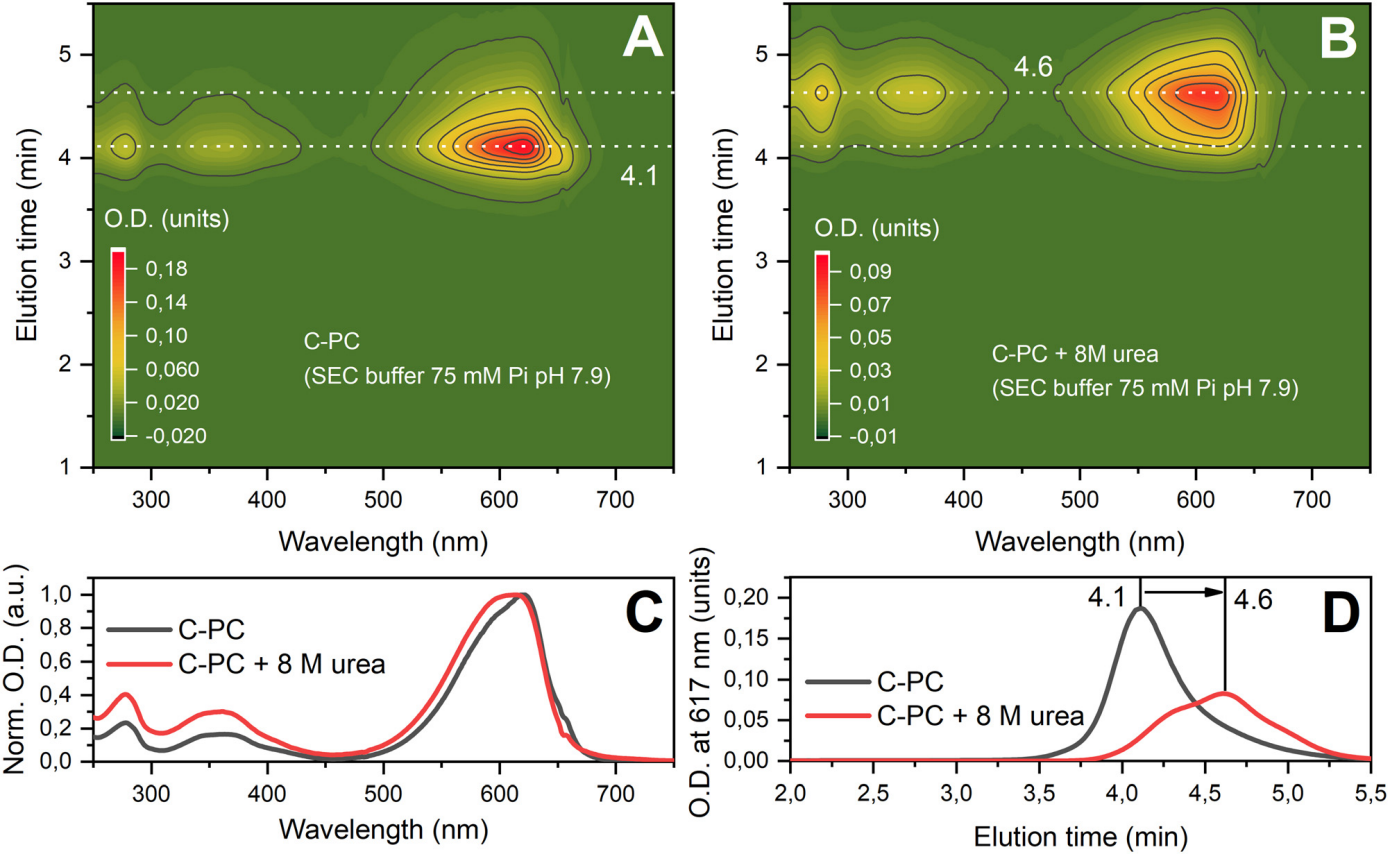
Hydrodynamic and spectroscopic characteristics of native and 8 M urea-treated C-PC. (a) and (b) Spectrally resolved chromatograms of C-PC solutions. The color indicates the optical density of the sample according to the legend. (c) Normalized absorption spectra of PC from the maxima of the elution profiles presented in panels (a) and (b) (4.1 and 4.6 min, respectively). (d) C-PC elution profiles from the experimental data presented in panels (A) and (B), obtained at 617 nm.

First, it is important for us to establish the structure of our C-PC samples in solution and to evaluate the possibility of structural changes under the influence of destabilizing factors. It is well known that urea in high concentrations leads to protein denaturation and disruption of the native oligomeric state of C-PC,^49^ which determines the mutual arrangement of chromophores in the pigment-protein complex. In crystal structures, C-PC of different species is represented by αβ_6_ hexamers, but according to our data obtained by SAXS, in solution C-PC is predominantly in a trimeric state (αβ_3_). This conclusion follows from the modeling based on the hexameric structure, which yields significantly larger errors compared to an approximation of the experimental x-ray scattering data with an αβ_3_ trimer. This is also supported by the relatively low value of estimated *MW* (Table S1), as well as by the *ab initio* molecular shape reconstruction by GASBOR, which agrees well with αβ_3_ trimer and is too small to accommodate αβ_6_ hexamer (Fig. 2). The disruption of the hexameric structure is probably due to the absence of linker polypeptides, which are removed at the stage of purification of C-PC, as evidenced by SDS-PAGE (supplementary Fig. 1^58^) revealing only two bands corresponding to the characteristic α and β subunit masses. It should also be noted that in our work, we used a buffer with a relatively low ionic strength (75 mM phosphate), while for working with intact phycobilisomes, a buffer with a phosphate content of at least 0.7 M is usually used, which is mandatory for maintaining the integrity of the phycobilisome core and the C-PC rod complex.^3,20,50–52^ Indeed, previous SAXS experiments on phycobilisomes dissolved in buffer lacking phosphate revealed a splitting of the phycobilisome rods into cylindrical subunits with a height of 28 Å, which represent trimeric phycocyanin.^53^ High concentrations of urea, on the contrary, have a chaotropic effect, which is thought to result in consistent disruption of protein–protein contacts between monomers within the αβ_3_ trimer and following denaturation of individual α/β subunits, which is confirmed by significant changes in the optical properties and energetic interactions between neighboring chromophores.^49^ Analysis of the SAXS data shows the decrease in the oligomeric state of C-PC at 8 M urea (see Materials and Methods and Table S1). It can be described as partial dissociation of trimers, which is in good agreement with SEC data [Fig. 3(d)]. The sizes of protein molecules at 8 M urea (d_max_ = 235 Å) exceed the sizes of ring-like trimers, but this issue can be described in terms of “unrolled” rings (supplementary Fig. 2^58^). Additional SAXS experiments for C-PC at 4 M and 6 M urea show a similar effect of partial unfolding to form particles in the 200–250 Å size range (supplementary Fig. 3^58^). However, upon a decrease in the urea concentration, C-PC refolds, and at least some protein–protein contacts are able to recover, which is clearly demonstrated by SEC (Fig. 3).

While native C-PC in SEC experiments is represented by a single symmetric peak at 4.1 min, its counterpart dialyzed against 8 M urea and loaded onto the same column equilibrated by urea-free buffer experienced a significant increase in the retention time to 4.6 min, reflecting a decrease in the molecular size (Fig. 3). Despite the relatively fast runtime (4–5 min), an urea-treated C-PC sample loaded onto the SEC column equilibrated without urea could experience partial reassociation of urea-disrupted C-PC oligomers. This resulted in the presence of several overlapping peaks in the elution profile [Fig. 3(d)]. Considering that C-PC as a result of renaturation partially returns to the state with initial hydrodynamic characteristics, we estimated the yield of such state as 9.6% (supplementary Fig. 4^58^). However, the average size of other oligomeric forms of the protein is significantly smaller (∼40 kDa) than that of the native trimeric C-PC (apparent *MW* ∼80 kDa; theoretical mass of (αβ)_3_ is ∼100 kDa). Thus, large aggregates formed at high concentrations of urea dissociate. We should also note the change in the optical properties of the protein after treatment with urea and partial reassociation [Fig. 3(c)]. A hypsochromic shift is observed in the S_0_-S_1_ absorption band, which can be attributed to disruption of the excitonic coupling between the C-PC chromophores.^49^ In the trimer, the chromophores associated with Cys-84 of the α subunit and Cys-82 of the β subunit are at distances suitable for such strong energetic coupling. We assume that contacts between (αβ)_1_ protomers can be disrupted during denaturation of C-PC and partially restored during protein renaturation.

Thus, the oligomeric structure of C-PC is mobile, capable of changing under the influence of environmental conditions. According to structural concepts based on the crystal structure of C-PC and structure modeling based on SAXS and SEC data in solution (Figs. 2 and 3), when the native oligomeric structure of C-PC is disturbed, a part of the chromophores might become exposed to the solvent and, accordingly, lose stabilizing environmental factors. The most likely candidates for the role of such chromophores are pigments located in the area of contacts of neighboring αβ_1_ protomers (see Fig. 1). Probably, the only covalent bond of the linear tetrapyrrole with the protein through the cysteine residue alone cannot preserve the planar structure of the phycobilin chromophore. When such chromophores are exposed to the solvent, rotation around single bonds becomes possible (see Fig. 1), which leads to a reduction in the effective conjugation length. Surprisingly, we found spectroscopic signatures of such states in C-PC even under non-denaturing conditions, indicating that stochastic disruption of the oligomeric structure of C-PC is possible in a part of the protein population.

**FIG. 4.**
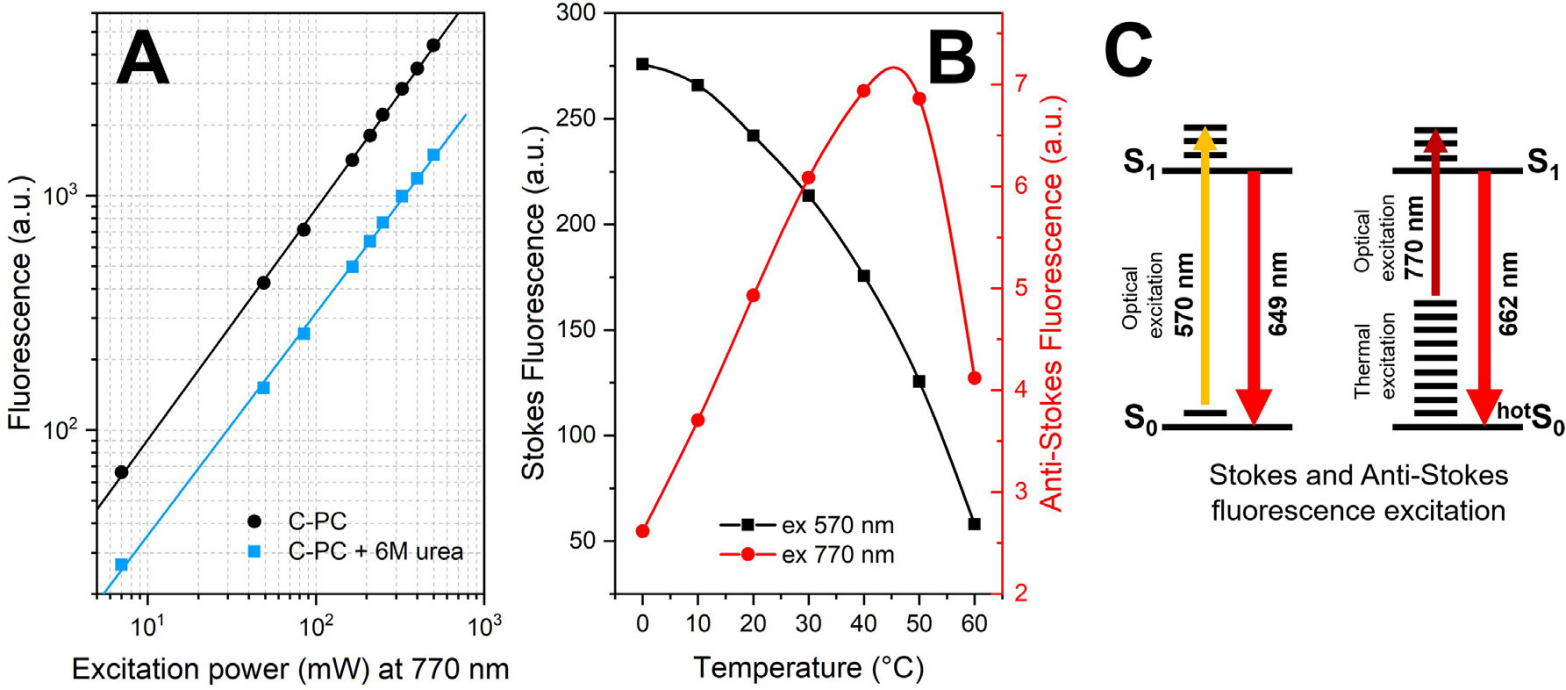
Mechanism of C-PC fluorescence excitation in the near infrared spectral range. (a) Dependence of time-integrated fluorescence intensity of C-PC in the range from 600 to 750 nm on the power of exciting light (770 nm). Note the logarithmic scale of fluorescence intensity and power. (b) Temperature dependence of Stokes (black line) and anti-Stokes (red line) fluorescence intensity of C-PC. (c) Schematic illustration of the principle of Stokes and anti-Stokes fluorescence excitation of C-PC using Jablonski diagrams.

When a C-PC solution was irradiated with near-infrared light (770–780 nm), anti-Stokes fluorescence in the 600–750 nm region was detected. The intensity of this signal was small compared to the fluorescence upon direct Stokes-type excitation into the absorption band of C-PC (approximately 620 nm). However, the use of single photon counters and a laser with a maximum power of up to 500 mW allowed us to record these signals with an excellent signal-to-noise ratio. First, we needed to verify whether this signal is a consequence of single-photon excitation of the C-PC chromophores, or whether two quanta can be absorbed due to the high power of the excitation light. To answer this question, we determined the dependence of the fluorescence intensity on the logarithm of excitation light power [see Fig. 4(a)]. In the range from 5 to 500 mW, the dependence is well described by a linear function with a slope equal to 0.99, which clearly indicates that this is a single-photon process within the range of laser powers used. Likewise, a parallel linear dependence is observed also for C-PC treated by 6 M urea, albeit it is significantly shifted to lower fluorescence intensities in the whole range of laser power values [Fig. 4(a)]. The low fluorescence intensity upon excitation in the 770–780 nm region is apparently due to the extremely low absorption of chromophores, which, in fact, cannot be measured using conventional spectrophotometers.

Next, we evaluated the effect of temperature on the fluorescence signal intensity. Figure 4(b) shows that when excited by high-energy quanta (570 nm), the signal intensity decreases with increasing temperature. This allows us to follow the process of C-PC denaturation, with the half-transition temperature equal to 59.6 °C (supplementary Fig. 1^58^). In contrast to the high-energy quanta excitation, when excited with low-energy quanta (770 nm), the C-PC fluorescence intensity increases up to 50 °C and decreases upon approaching the melting temperature of the protein (around 60 °C). An increase in temperature reduces the quantum yield and fluorescence intensity by increasing the probability of a nonradiative excited state decay. However, with a temperature increase, the population of higher vibrational sublevels also increases, which eventually enables a transition to an electronically excited state upon absorption of a quantum, even if its energy is less than the energy of the 0–0 transition [see Fig. 4(c)]. Thus, the energy of the environment provides the possibility of an optical excitation of C-PC chromophores by quanta with low energy of about 1.6 eV, which is 25% less than the energy corresponding to the absorption maximum of C-PC (2.0 eV), just in terms of a molecular heat pump. Thus, the emission of phycocyanin upon excitation at 770–780 nm is represented by anti-Stokes fluorescence. The consequence of such an excitation scheme is the possibility to specifically for a certain subset of chromophores, which are closely interacting with the solvent environment, since their state is energetically different from the bulk chromophores.

**FIG. 5.**
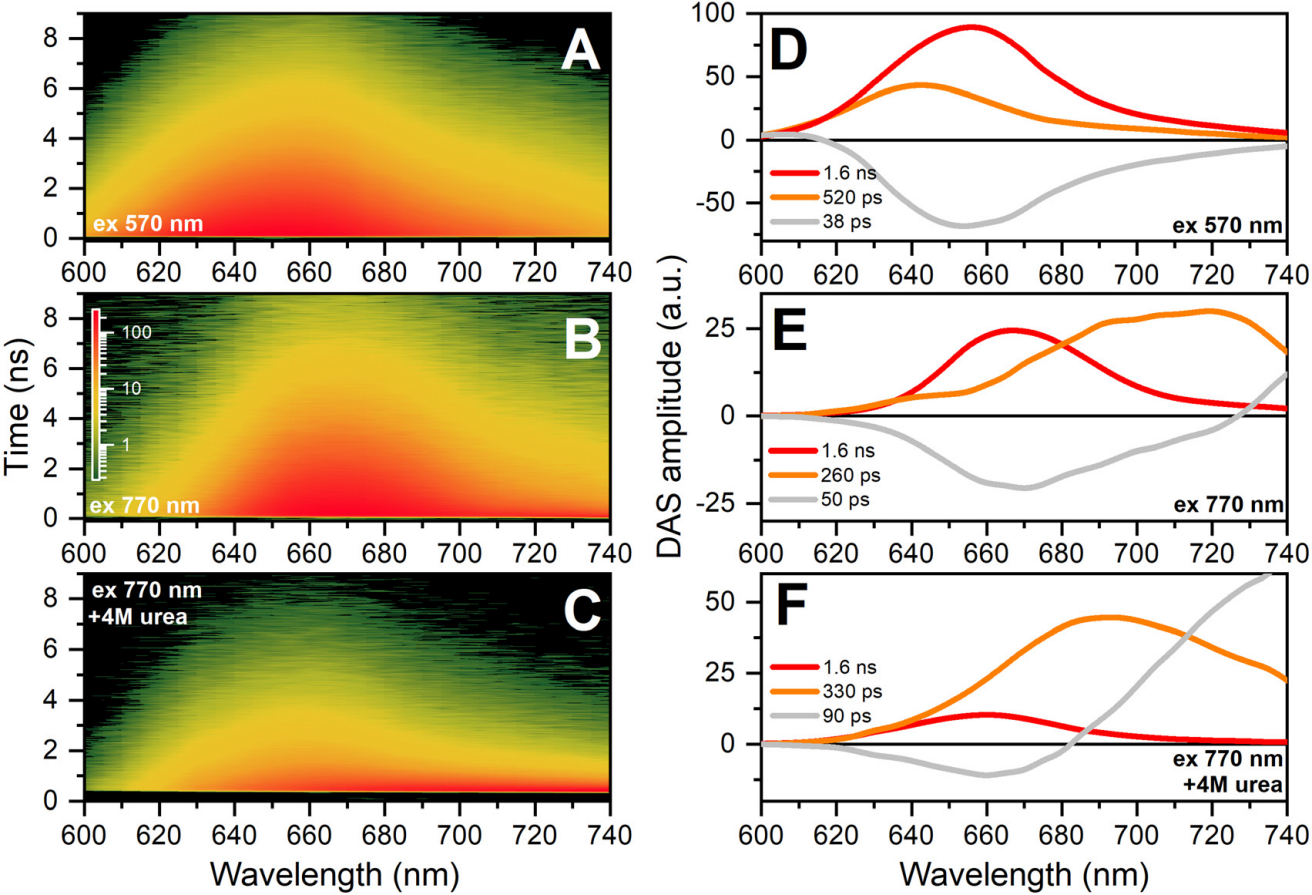
Time-resolved spectra of Stokes (**a**) and anti-Stokes (b) and (c) C-PC fluorescence excited by femtosecond laser pulses at 570 and 770 nm (3.4 and 200 mW/cm^2^, respectively). The fluorescence intensity is shown in color code, and the logarithmic color scale common to all three figures is shown in panel (b). Decay-associated spectra (DAS) of phycocyanin fluorescence in the Stokes (d) and anti-Stokes (e) and (f) regimes. The numbers show characteristic lifetimes of excited states derived from the global analysis of fluorescence decay kinetics measured in the range from 600 to 740 nm.

To describe the properties of C-PC chromophores that are excited by the absorption of low-energy photons, we measured time-resolved fluorescence spectra and performed global analysis of the kinetic components of these spectra (Fig. 5) to determine the decay-associated spectra (DAS). Global analysis of the fluorescence decay kinetics shows that upon excitation at 570 nm, the main population of C-PC chromophores is a form with a lifetime of 1.6 ns, and the emission maximum of the spectrum is red-shifted by about 5 nm compared to the position of the maximum of the steady-state fluorescence spectrum. However, the existence of a shorter wavelength component (640 nm) with a low amplitude and a lifetime of about 520 ps should be noted. As it was already noted, native C-PC trimers are characterized by energetic coupling of neighboring chromophores, between which both excitonic and resonant transfer of electronic excitation energy are possible and occur rapidly due to the short intramolecular distances. In the case of C-PC, energetic coupling can be manifested as a delayed rise kinetics of fluorescence after the completion of direct optical excitation, which is reflected in the form of components with negative amplitude in decay-associated spectra (DAS). Upon excitation of the anti-Stokes fluorescence of C-PC [Figs. 3(b) and 3(e)], we also detected a component with a characteristic lifetime of 1.6 ns, but its maximum was shifted to 665 nm. However, in the case of anti-Stokes fluorescence, the main contributing component of the kinetics was characterized by a lifetime of about 260 ps dominating in the 700–720 nm region. Such a short lifetime and the position of the emission maximum are uncharacteristic for phycobiliprotein chromophores in the native state.^29^ However, it is known that chromophore isomerization as a result of protein denaturation can be accompanied by similar changes in spectral characteristics.^11,54–56^ In line with this, high concentrations of urea (and other denaturing agents and factors) lead to disruption of the native oligomeric state of phycobiliproteins, which is accompanied by a decrease in the fluorescence quantum yield due to increased conformational mobility of the linear tetrapyrrole chromophore.

Therefore, to test the hypothesis that the rapidly decaying state in the anti-Stokes fluorescence of phycocyanin may be a signature of chromophores with altered geometry, we performed experiments with C-PC in the presence of 4 M urea [Figs. 5(c) and 5(f)]. We found that in the buffer containing 4 M urea and 770 nm excitation, the yield of the chromophore state with a lifetime of 1.6 ns decreases significantly, while the main contribution to the fluorescence is made by rapidly decaying states with emission in the 680–720 nm region. Stokes fluorescence of phycocyanin in the presence of 4 M urea differs insignificantly from the protein fluorescence under non-denaturing conditions [Fig. 3(a)]. Thus, high-energy quantum absorption (570 nm) leads to the occupancy of excited states of chromophores with native conformation, even in the presence of 4 M urea. However, excitation at 770 nm predominantly populates excited states of chromophores with an altered configuration, and denaturation of the protein contributes to an increase in the yield of these states [see Figs. 5(e) and 5(f)]. This indicates possible differences in the absorption efficiency of the native and altered chromophore states in the red and infrared ranges.

**FIG. 6.**
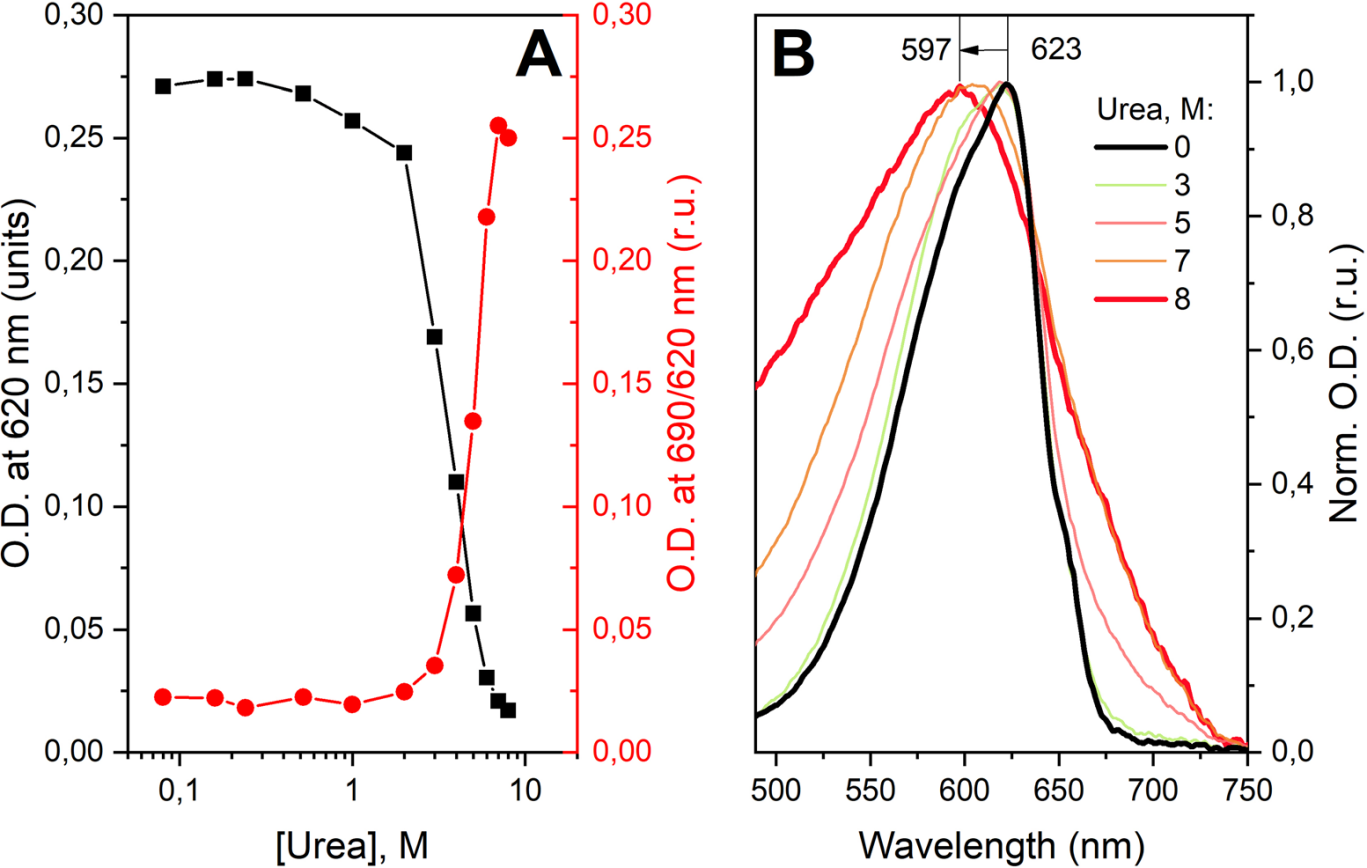
Effect of urea concentration on the absorption spectrum of C-PC chromophores. (a) Dependence of optical density at 620 nm (black squares) on urea concentration and normalized to the maximum absorbance at 690 nm (red circles) at 25 °C. (b) Absorption spectra (normalized to maximum) of phycocyanin solutions containing different concentrations of urea.

We analyzed changes in C-PC absorption spectra in solutions with increasing urea concentration from 0 to 8 M (Fig. 6). Up to 2 M urea, the absorption in the red region changes insignificantly, although a hypsochromic shift of S_0_-S_1_ is observed. At higher concentrations of urea, the intensity of absorbance in the S_1_ region decreases significantly [Fig. 6(a)], while absorbance in the 350 nm region increases (data not shown), these effects are well known and have been described in numerous papers.^11^ However, important for describing the anti-Stokes fluorescence of C-PC is the significant broadening of the S_1_ absorption band, which not only compensates for the hypsochromic shift but also significantly increases the probability of light absorption in the region above 690 nm [Fig. 6(b)]. Thus, the higher yield of red-shifted forms with short lifetimes in the solution of C-PC with 4 M urea (Fig. 5) is probably due to an increase in the concentration of chromophores with high (compared with the native) absorption in the near-infrared region.

**FIG. 7.**
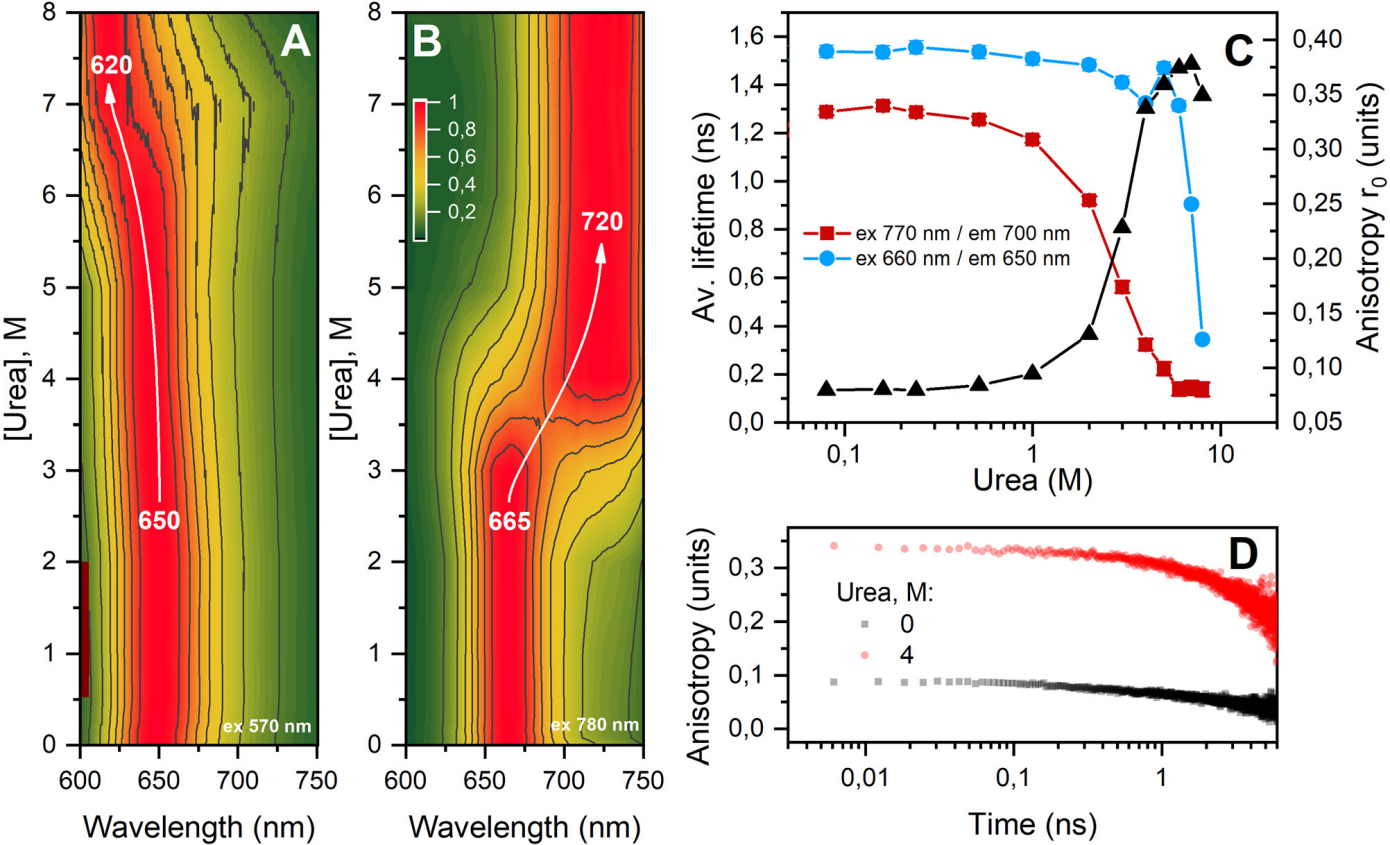
Normalized time-integrated Stokes (a) and anti-Stokes (b) fluorescence spectra of C-PC solutions containing different concentrations of urea at 25 °C. The color indicates the normalized fluorescence intensity according to the color scale shown in panel (b). Stokes fluorescence was excited by 150 fs laser flashes at 570 nm (2 mW/cm^2^), anti-Stokes fluorescence was excited at 780 nm (400 mW/cm^2^). Arrows indicate the direction of the shift of the emission peak. For all fluorescence measurements, the protein concentration in the solution was approximately 65 nM, which provides an optical density of less than 0.1 units at 620 nm. (**c**) Dependence of the average fluorescence lifetime of C-PC chromophores on urea concentration during excitation at 770 nm (red squares) and 660 nm (blue circles). The right Y-axis shows initial values of fluorescence anisotropy r_0_ (black triangles). (d) Characteristic relaxation kinetics of C-PC fluorescence anisotropy in the absence (black dots) and presence of 4 M urea (red circles). Fluorescence was excited at 660 nm and recorded at 650 nm. The experiment was conducted at 25 °C.

The analysis of time-integrated (steady-state) fluorescence spectra of C-PC solutions measured under 570 nm excitation revealed a transition between the main chromophore excited state (650 nm) and the short-wavelength form of it (620 nm) at urea concentrations of 6 M and above (Fig. 7). When anti-Stokes fluorescence is excited, a transition between the chromophore forms with emission maximum at 665 nm and 720 is observed already at 4 M urea. Thus, the anti-Stokes fluorescence of C-PC chromophores excited at 770–780 nm is more sensitive to changes in the state of the protein environment, which is manifested by changes not only in the position of the emission maximum but also in the average fluorescence lifetime [Fig. 7(c)]. The magnitude of the quantum energy probably plays a decisive role in the settlement of the different states of the chromophores, since the excitation of fluorescence at 660 nm [Fig. 7(c)] reveals dependency of average fluorescence lifetime similar to excitation at 570 nm (data not shown). At the same time, the anisotropy of C-PC fluorescence excited at 660 nm was sensitive to changes in the oligomeric state of PC, which correlates with the data on the lifetime of anti-Stokes fluorescence excited at 770 nm [Fig. 7(c)]. At high concentrations of urea, the observed values of intrinsic anisotropy $r_{0}$ are close to a theoretical maximum for single-photon excitation (0.4), which corresponds to a parallel orientation of the excitation and emission dipoles. In contrast, lower $r_{0}$ values are observed in the native state of the protein, which can be attributed to a significant contribution of energetic coupling between neighboring chromophores (see Fig. 1). Obviously, excitonic coupling and resonance energy transfer must be disrupted as the distance between the chromophores increases during the transition of native C-PC trimers (αβ_3_) to monomers (αβ_1_). Our results are in good agreement with the data of non-linear polarization spectroscopy in the frequency domain, which showed that chromophores α84 and β84 are not excitonically coupled in C-PC monomers.^57^ Thus, we believe that spontaneous or induced disruption of protein–protein interactions between C-PC αβ_1_ monomers leads to an increase in conformational mobility and chromophore isomerization. It should be noted that high urea concentrations affect the viscosity of the solution, so the assessment of the hydrodynamic mobility of C-PC by anisotropy relaxation kinetics [Fig. 7(d)] does not seem informative. However, it should be noted that these kinetics are not monoexponential, and the subnanosecond component should be probably attributed to the mobility of the chromophore.

## IV. CONCLUSIONS

The stability and brightness of C-PC account for its popularity for fluorescence labeling and imaging purposes. However, analysis of the spectral characteristics of C-PC shows that its chromophores can be in states with significantly different fluorescence quantum yield and position of emission maximum. In this work, by combination of spectroscopic and structural methods, we characterized four distinct states of C-PC chromophores (emitting at 620, 650, 665, and 720 nm, see Fig. 7) and assigned the fast component in the anti-Stokes fluorescence decay kinetics in the range of 690–750 nm to chromophores with an increased conformational mobility, which is likely present in proteins with abnormal or disturbed contacts between αβ protomers. It is known that increasing the concentration of urea in the C-PC solution consistently leads to a change in the oligomeric state of the complex from hexamer (or trimer) to monomer and, finally, individual α and β subunits.^29,54^ We propose that a change in the oligomeric state of C-PC causes exposure of phycocyanobilin chromophores, providing additional levels of conformational freedom. However notably, we were able to show that such chromophores exist even under non-denaturing, i.e., physiological conditions (Fig. 5). Although the fraction of such states is small compared to the total number of chromophores, their broad absorption spectrum, which extends far into the red and infrared regions, allowed us to observe such red-shifted states upon fluorescence excitation in the near-IR. Unlike bulk chromophores, these red states are sensitive to the conformational dynamics of the protein, which opens novel possibilities for the investigation of protein–protein interactions and protein stability. Since C-PC interacts with a variety of structural and regulatory proteins in cyanobacterial antenna complexes, we believe that anti-Stokes fluorescence may reveal important aspects of these interactions. States of phycocyanobilins with sub-nanosecond lifetimes could serve as excitation energy traps necessary for processes involved in photoprotection. Alternatively, red states may expand the range of wavelengths, which can be used to perform photosynthesis close to or even beyond the famous “red limit.” The molecular heat pump mechanism could also be important for thermophilic cyanobacteria to provide them with enhanced photosynthesis efficiency. We suppose that these possibilities should be explored in the future.

We also propose that the phenomenon of anti-Stokes fluorescence of C-PC can potentially be used to develop optical imaging methods for visualization of environmental parameters. For example, the data presented in Fig. 4(b) show that anti-Stokes fluorescence not only is potentially a convenient tool for determining the thermodynamic stability of a fluorescent protein but can also be used to determine local temperature. Indeed, the concentration of special states of chromophores and the associated anti-Stokes fluorescence quantum yield depend on temperature, which facilitates to determine the corresponding dependence and solve the inverse problem. The most promising method for these purposes seems to be fluorescence lifetime imaging microscopy (FLIM) in combination with single-photon excitation in IR, which would simultaneously permit to detect the fluorescence decay kinetics and to determine the contributions of the components in different parts of the sample.

## Data Availability

The data that support the findings of this study are available from the corresponding author upon reasonable request.
